# Nervous Influence on Salivary Secretion

**Published:** 1868-12

**Authors:** 


### SELECTED ARTICLES.

### ARTICLE V.

### *Nervous Influence on Salivary Secretion.*

Whilst great attention has for many years been directed to the mode in which the nerves distributed to muscle terminate, and endless disquisitions have appeared on the methods by which their functional activity may be excited or modified, comparatively few investigations have been made on the ultimate ramifications of the nerves distributed to glands, or on the the effects of nervous excitation on the amount and quality of the secretion they severally produce. The recent extended and careful researches of Pfuger, Schiff, and Heidenhain on the salivary glands, however, which may be regarded as a continuation of those previously made by Bernard and Ludwig, bid fair to take away this reproach, and to give a clearer insight into this obscure domain of physiology. Of all the glandular organs, none are so well adapted for experimental investigations as the submaxillary and the parotid glands. Their superficial position—permitting them to be easily exposed without serious injury being inflicted on the animal;—their limited and well-known nervous supply, and the facility with which by the introduction of canulæ into their excretory ducts variations in secretory activity consequent on excitation of their nerves may be recognized,—are all circumstances which contrast favourably with the difficulties encountered in examining such organs as the liver or pancreas, and have led to the selection of the former glands for experiment by the later observers. A few years ago it was generally admitted that the influence of the nervous system upon a gland was not so much exercised upon the process of secretion, which was supposed to proceed continuously, as upon the excretory act, the induced contraction of the muscular walls of the ducts being supposed to express the secretion which had accumulated in their interior. The observations of Stilling and Ludwig rendered some modification of this view reqisite, showing as they did the great influence the nerves possessed over the blood-vessels, augmenting their calibre, and increasing the flow of blood through them and the pressure under which it moved. They erred, however, in attributing the dilation observed in the arteries to a remora of the blood consequent on contraction of the small veins. This mistake was corrected by the observations of Schiff and Bernard, who showed that the veins, so far from contracting when the cerebro-spinal nerves were irritated, dilated, conveyed a fuller current of blood (which assumed a bright scarlet tint,) and sometime pulsated, whist the pressure of the blood in their interior was also decidedly increased. Opposite effects were shown to be produced by irritating the sympathetic, while similar effects followed the paralysis of that nerve. It was therefore admitted that the contraction of the vessels was under the control of the sympathetic nerve, whilst the cerebro-spinal nerves in certain instances could effect an *active dilation* of the vessels ; and the difference in the quantity of the saliva secreted were believed to be due to the variations in the pressure of the blood in the vessels. Eckhard, however, soon showed that, besides a diff-ference in the quantity, there was a marked difference in the *quality* of the fluid secreted by the submaxillary gland according to whether the chorda tympani or the sympathetic nerves were irritated : in the former case the fluid being thin and watery; in the latter thick, viscid, and slightly turbid, from the presence of numerous particles of protoplasm possessing the power of spontaneous movement. The parotid was less easy to experiment upon, and some difference of opinion existed in reference to it, Eckhard believing that in the sheep it secreted continously, and was influenced by either the cerebro-spinal or sympathetic nerves, whilst Von Wittich held that the activity of the gland could be energetically roused through the sympathetic. The subject was taken up by Pfluger at this point, by whom the salivary glands were subjected to most careful microscopic scrutiny, especially in reference to the mode of termination of the nerves. He ascertained the important fact that the nerves are in direct continuity with the gland-tissue, through the intermediation of the secreting cells which line the alveoli and the ducts, and that fine branches, apparently both of the cerebro-spinal nerves and of the sympathetic, may be traced into the nuclei of the cells, sometimes being fine and singly contoured, sometimes varicose, and sometimes ending in a large multicaudate ganglion cell, the branches of which enter the secreting cells, and terminate, like the former, in the nuclei, which in fact appear as clavate expansions or terminations of the nervous fibres. The observations of Pfluger have been interestingly supplemented by those of Heidenhain, who believes he has been able to show that in man and the dog, though not in the rabbit, two forms of secreting cells are found in the alveoli—one in which the cells are soft, ill-defined masses of protoplasm or germinal matter, with round nuclei and finely granular contents ; the other in which the cells have a well-marked cell-wall, transparent contents, not easily tinted with carmine, and flattened nuclei. The former appear to produce watery saliva, containing ptyalin, the latter a secretion containing a large proportion of mucus. The result of prolonged irritation applied either to the sympathetic or to the chorda tympani caused an increase in the quantity of the saliva, though in the former instance it contained a larger proportion of the amœboid particles and mucin, and in the latter saliva of a more watery character, with ptyalin. As a general result of his experiments, he considers there is strong evidence that irritation of certain nerves may induce lively cell-formation and metamorphosis. Surely these facts are replete with interest, and lead us a step nearer to the comprehension of the intimate connexion that exists between the nervous system and all parts of the organic framework. And we can no longer be surprised at the powerful influence exerted by the nerves over glands; as instance of which the alteration that occur under various conditions in the secretion of the mamary gland during lactation, in the functions of the liver and the kidneys, may be referred to, since it may reasonably be concluded that arrangments analogous, if not identical, with those just described exist in all these glands.

In conclusion, we may just remark that Schiff's observations on the nervous supply of the parotid, which has been much disputed, are of great interest. He finds it is derived in part from the seventh, through the temporo, and cervico-facial branches; in part from the auriculo-temporal of the fifth, and the auricular branch of the cervical plexus; and, lastly, from the sympathetic. Its most important branches are probably derived from the facial through the lesser superfacial petrosal nerve, which enters the otic ganglion, and is distributed by the branches of that ganglion to the gland. These excite it to action, or constitute its motor branches. It would of course be a fact of the greatest importance if it could be established that particular nerves were distributed to special forms of cells; but at present there is no evidence to warrant this conclusion.—*Lancet.*

